# Household financial literacy and relative poverty: An analysis of the psychology of poverty and market participation

**DOI:** 10.3389/fpsyg.2022.898486

**Published:** 2022-07-22

**Authors:** Shanping Wang, Peng Cao, Shao Huang

**Affiliations:** ^1^Business School, Hunan Normal University, Changsha, China; ^2^School of Mathematics and Statistics, Hunan Normal University, Changsha, China; ^3^School of Electronic Information, Hunan First Normal University, Changsha, China

**Keywords:** financial literacy, relative poverty, psychology of poverty, mechanism analysis, market participation

## Abstract

Financial literacy is the significant human capital factor affecting people's ability to obtain financial services. Evaluating the relationship between financial literacy and relative poverty is of great significance to poverty reduction. This study investigated the impacts of financial literacy on relative poverty from the perspective of poverty psychology and market participation using data from the 2017, 2019 China Household Finance Survey (CHFS). The empirical findings showed that financial literacy can alleviate relative household poverty through household participation in entrepreneurial activities, commercial insurance participation and the choice of lending channels. Financial literacy has significant poverty reduction effect on households of continuous operation, reduces the likelihood of exiting operation. Further discussion showed that the poverty reduction effect of financial literacy is more pronounced among households with higher levels of financial literacy, under the age of sixty, low levels of indebtedness and in the eastern region. Our study provides empirical evidence for encouraging market participation and promoting financial literacy and provide valuable recommendations for the policymaker to improve poverty reduction effect in the developing country context.

## Introduction

Poverty has been a top social issue in the world, and the 2030 Agenda for Sustainable Development was officially adopted at the United Nations Sustainable Development Summit, where “eradicating poverty in all its forms” is the first of many goals (Tollefson, 2015; Li et al., 2016). However, the issue of poverty is very complex. With the expansion of the concept and application of poverty academics have gradually shifted from a focus on absolute poverty to relative poverty. Absolute poverty generally refers to a household income is insufficient to maintain minimum subsistence conditions, falling into absolute material deprivation. Unlike absolute poverty, relative poverty refers to people living below the average level of other groups in society. The problem of relative poverty is somewhat complex and dynamic (Wan et al., 2021). First, relative poverty is related to the absolute poverty line set by countries and regions. If the poverty line of the World Bank is taken as the standard, China will still have large number of poor people. Second, the development trend of poverty is dynamic. Solving the problem of relative poverty not only focuses on the income of poor groups, but also pays attention to cultivating the endogenous development capacity of poor groups (Capacity Development Group, 2006). Therefore, how to continue to effectively promote poverty governance and cultivate the endogenous development motivation of poor households, so that households can sustainably increase their income and get rid of poverty is an important issue for the future governance of poverty.

Financial literacy, which is an important human capital factor, specifically refers to people's comprehensive ability to master the basic economic knowledge and financial concepts to manage and allocate funding resources to achieve household benefits through financial services (Atkinson and Messy, 2011). A new financial supply system has been developed through the close combination of Internet communication technologies and financial supply, which improves the breadth of financial coverage and the innovation of financial services goods (Reboul et al., 2021). Families with a certain level of financial literacy can obtain development opportunities under the background of digital finance and play a crucial role in the governance of relative poverty. However, according to the survey data, the financial literacy of residents in many countries and regions in the world is generally low. For example, Disney and Gathergood (2013) found in the UK household survey questionnaire database that the questionnaire contained three questions on simple interest, compound interest and minimum repayment, and the results showed that 11% of the respondents got all the answers wrong and only 30% got all the answers right. Klapper et al. (2013) found that Russia, as a country with rapid growth in consumer lending, only 41% of the respondents could understand the retaliatory interest, and 46% could answer the simple concept of inflation. Lusardi and Tufano (2015) used the sample of the United States to suggest that only 1/3 of the respondents have a certain understanding of the calculation of compound interest and the details of the use of credit cards. The Brief Report on Consumer Financial Literacy Survey (2021) published by The People's Bank of China shows that,^1^ the financial demand and their financial literacy of Chinese consumers are improving. At the same time, the report points out that residents' expectations of financial investment are also irrational, which is prone to irrational investment behavior. If residents have high financial literacy, they can better understand bank lending policies, insurance services and other related financial services, reducing the cost of financial services (Van Rooij et al., 2011). When households have access to certain funds and insurance services, they can smooth consumption, participating in Entrepreneurship and lower poverty risk shocks, thereby alleviating poverty. Therefore, the level of financial literacy is directly related to whether households can grasp the income opportunities brought about, thus having an impact on their current livelihood status. This provides a new idea for solving the problem of relative poverty, which is important to improve the quality of poverty reduction and promote regional sustainable poverty alleviation.

Existing research have investigated and achieved various conclusions on ways to increase household income and alleviate relative poverty. From the macro perspective, existing scholars believe that the “Trickle-down effect” of economic development (Dollar and Kraay, 2002; Yang et al., 2021), inclusive financial development (Ho and Iyke, 2017; Kong and Loubere, 2021), labor mobility, land transfer (Carvalho et al., 2016; Li et al., 2020), infrastructure construction and urbanization (Chen et al., 2019; Medeiros et al., 2021) and other factors alleviate relative poverty. However, studies have also point that while economic growth can explain the decline in poverty rates, it has poor explanatory power and there is no evidence that such growth can spontaneously reduce incidence of poverty (Kakwani and Pernia, 2000). In addition, some studies have emphasized the relationship between financial development and poverty, but the findings have not been consistent (Bolarinwa and Akinbobola, 2021). It is worth noting that the above studies analyze the impact of external conditions such as policy implementation and macro environment on relative poverty, ignoring the subjective initiative of poor subjects and failing to consider the role of human capital inherent in poor subjects. Although studies have analyzed poverty alleviation of the poor from human capital factors such as education, health, and work experience (Zon and Muysken, 2001; Quinn, 2006; Bellemare and Bloem, 2018; Liu F. et al., 2021), little literature has examined household poverty reduction from the perspective of financial literacy.

Some studies have shown that financial literacy has a certain positive impact on the subjective willingness of actors. On the one hand, households with higher financial literacy can enhance the inclusiveness of inclusive finance (Grohmann et al., 2018). For instance, increasing access to banking business and microfinance information can improve the availability of financial services (Hasan et al., 2021). There are significant effects on household consumption level and consumption inequality (Dinkova et al., 2021; Koomson et al., 2021), family members' retirement plans (Lusardi and Olivia, 2007), and household entrepreneurial behavior (Zhao and Li, 2021). On the other hand, financial literacy has a positive effect on financial behavior, such as asset allocation choices (Lusardi et al., 2013), financial market participation (Van Rooij et al., 2011; Nguyen and Nguyen, 2020), financial decision-making (Guiso and Tullio, 2008), investment diversification (Guiso and Jappelli, 1998), reduction of over-indebtedness (Lusardi and Peter, 2009), and credit demand (Lusardi and Peter, 2009; Stango and Jonathan, 2009). Therefore, improving the financial literacy of family members in their daily participation in production and business processes can make rational and optimal economic decisions to obtain benefits and reduce the incidence of poverty (Lusardi et al., 2013; Emara and Mohieldin, 2020). Therefore, one aim of this study is to examine the effect of financial literacy on the relative poverty of households.

Improving household financial literacy and achieving poverty alleviation are the results of a combination of economic decisions. Existing literature has studied the improvement of household financial literacy and absolute poverty alleviation from the perspective of household rationality. It is found that financial literacy has a positive impact on alleviating income poverty and asset poverty in rural households (Xu et al., 2021). However, there are also literature showing that households with higher financial literacy have a greater likelihood of increasing leverage through financial instruments and overdraft consumption; if the prices of financial assets falls sharply and the overdraft is too large, they will fall into poverty for a long time (Sarthak and Ashish, 2012). Most believe that the poverty reduction effect is generated through the rational asset allocation choices of actors (Shan, 2019), but did not consider the cultural factors behind financial behavior, i.e., households living in poverty for a long time, after being influenced by some culture, will generate poverty dependence, conservative risk preferences, information barriers and other psychological perceptions of poverty thus triggering severe financial needs of households and self-inhibiting phenomenon of market participation, thus falling into the poverty trap. Therefore, the second purpose of this study is to analyze the internal mechanism of the influence of financial literacy on relative poverty from the perspective of poverty psychology and planned behavior, test the mediating effect and theoretically expand the understanding of the impact mechanism of relative poverty.

Although previous research had confirmed that financial literacy can contribute to poverty alleviation (Shan, 2019; Xu et al., 2021), this effect is heterogeneous in different household characteristics and regions. Different household characteristics can have an impact on households' financial market participation (Azeem et al., 2017; Decerf, 2017). Younger household members are more likely to learn basic financial knowledge, cross the consumption threshold of financial services, and be more receptive to financial services and better able to enjoy the benefits of financial development than older people (Calvin et al., 2018). Households with higher indebtedness tend to have higher financing constraints and may engage in more irrational economic behavior, making it difficult for households to get rid of poverty quickly (Sarthak and Ashish, 2012). Therefore, the third aim of this study is to explore the heterogeneous effects of financial literacy on relative poverty under different household characteristics and regional development levels.

Compared with the existing literature, three contributions to this analysis can be summarized here. First, taking financial literacy as the main human capital factor affecting households' relative poverty enriches the literature exploring the relationship between the two in empirical studies. Some literature believes that family members with certain financial literacy can rationally allocate assets and produce poverty reduction effect from a rational perspective. However, for households living in poverty for a long time, there is a certain degree of poverty psychological cognition. Whether the improvement of household financial literacy can inhibit the occurrence of relative poverty or not is lack of relevant research. From the perspective of poverty psychology theory, our study constructs a theoretical system of financial literacy poverty reduction, which helps to explain how to improve household financial literacy to alleviate the relative poverty. Second, to reveal the inner mechanism of household financial literacy to alleviate relative poverty. Based on the theories of poverty psychology and financial behavior, we verify the role of micro-mechanisms of household financial market participation in financial literacy poverty reduction, provide new ideas for guiding residents to participate in financial markets and thus alleviate relative poverty. Third, we analyze the heterogeneity of the financial literacy on relative poverty, in different household characteristics and regions, and clarify put the boundaries within which the results of our study are valid. In addition, we also hope that this study can help relevant departments to formulate management countermeasures for enhancing residents' financial literacy and stimulating household residents' financial market participation, achieving new breakthroughs in financial literacy poverty reduction.

The remaining sections of this paper are arranged as follows. Section Theoretical background and hypothesis development presents the theoretical link between financial literacy and relative poverty. Section Methodology discusses the methodology, which includes data sources, measurement of key variables and estimation techniques. Section Empirical results and analyses provides contains the findings and analyses, including baseline regression results, endogeneity treatment and robustness tests. Section Analyses of impact mechanisms presents the analyses of impact mechanism and the further analysis. Section Discussion provides discussion and Section Conclusion presents conclusion.

## Theoretical background and hypothesis development

When the family has certain material conditions, there has been a shift in poverty governance from absolute poverty to relative poverty. Absolute poverty is usually defined as the difficulty in obtaining income or necessities to meet the basic survival of a household, and the failure to secure basic needs such as housing, utilities, transport, compulsory education and basic health care, relative poverty is identified by setting a poverty line, i.e., 50 or 60% of the median household income. On the other hand, according to Sen (1999), the main causes of poverty are The deprivation of the household's power and ability to access benefits. Relative poverty is connoted by the term “moderate poverty”, which refers specifically to the difficulty of the household to reach a socially acceptable level due to a lack of social resources and the ability to develop itself (Wu, 2021). In the process of alleviating relative poverty, poverty alleviation efforts should focus on building an anti-poverty path based on the capacity building and resource accumulation of households. Therefore, the governance of relative poverty should also start with improving the viable capacity of households to access economic and social development opportunities and thus escape poverty.

**Figure 1:**
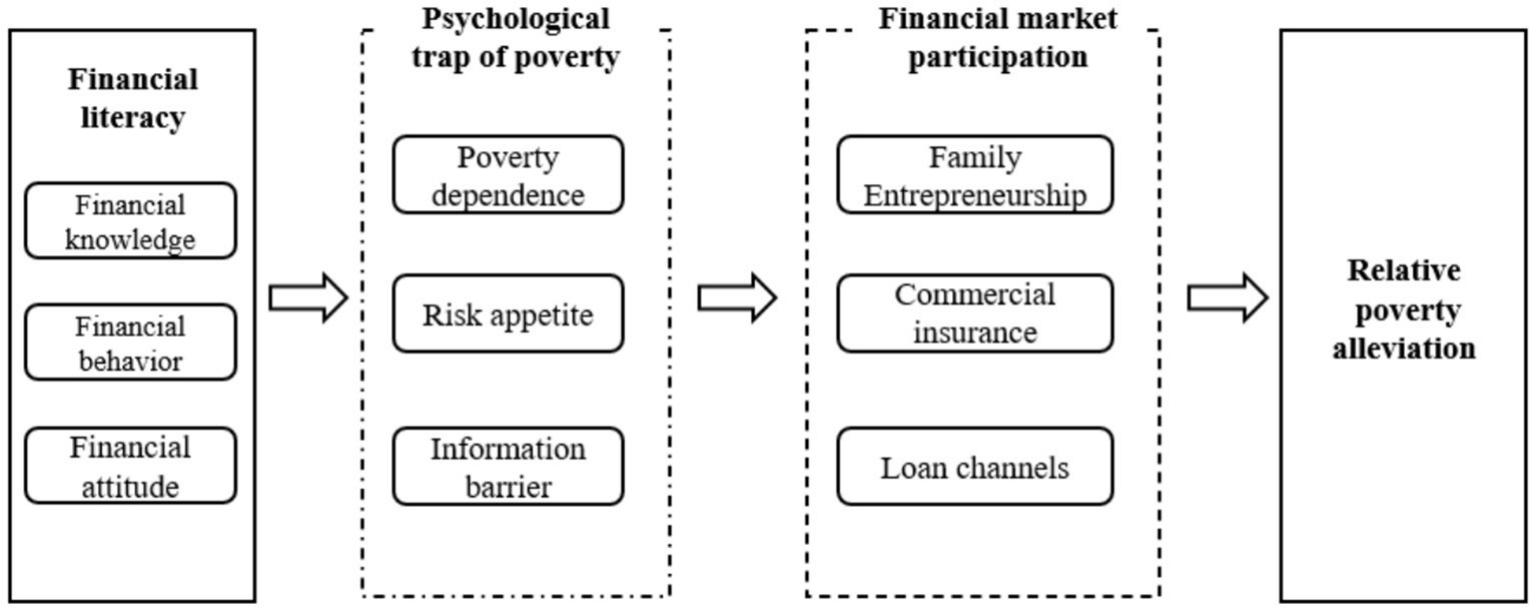
Mechanism of household financial literacy alleviating relative poverty.

It has been shown that the emotional state, social pressure, and other psychological characteristics of poor groups can influence household economic behavior (Guiso and Paiella, 2008; Carvalho et al., 2016; Haq et al., 2021). The theory is generally explained from two perspectives: the behavioral economics and the poverty psychological. The theory of behavioral economics argues that liquidity constraints or background risks under imperfect formal financial markets are seen as an explanatory theoretical mechanism by which poverty affects economic behavior (Gennetian and Shafir, 2015; Key, 2022). While the poverty psychological theory argues that chronic poverty states may lead individuals to develop psychological characteristics such as negative affect and stress, resulting in poor groups exhibiting poverty dependence due to a lack of initiative, lack of social responsibility. Psychological trap of poverty is that this psychology of poverty will lead to insufficient household participation in financial markets and is not conducive to poverty alleviation (Haushofer and Fehr, 2014; Fu et al., 2020). Therefore, this paper argues that financially literate can alleviate relative poverty through household participation in entrepreneurial activities by reducing “Poverty dependence”, household commercial insurance participation by improving “Risk appetite”, and household credit access by breaking down “Information barrier” (see Figure 1). Therefore, this paper proposes hypothesis 1:

**Hypothesis 1:** Financial literacy can alleviate relative poverty.

The poorer groups are less resource endowed and less able to develop themselves, and in the long run tend to develop a negative dependency mentality. Families with high financial literacy will the ability to use their knowledge and skills to make reasonable and effective decisions in management of money and resources, have entrepreneurial skills to circumvent negative dependency (Evans and Jovanovic, 1989; Santos et al., 2018).

According to the Theory of Planned Behavior, behavioral intention is a direct determinant of behavioral implementation, and individual behavioral attitudes and perceptual behavioral control influence individual behavioral intention at different levels (Yang et al., 2020). Entrepreneurial intention is a visual representation of the subjective attitudes of potential entrepreneurs regarding entrepreneurial activities and can effectively predict the probability of entrepreneurial behavior. Therefore, entrepreneurial behavior is subject to entrepreneurial intentions, and subjective norms formed by attitudes about investment, financing and risk management behaviors, perceptual behavioral control and perceptions of support and pressure in the external environment combine to influence their entrepreneurial intentions.

Financial literacy can promote household entrepreneurial activity to alleviate relative poverty through the following pathways. First, financial literacy has an impact on household entrepreneurial activity according to the framework of the theory of planned behavior. Households choose entrepreneurial options based on a direct measure of the benefits, costs, and risks. At the same time, entrepreneurship as a form of risky investment can have a direct exclusionary effect on households with low endogenous developmental dynamics and weak human capital. Therefore, financial literacy has a direct impact on individual financial market participation and allocation decisions to different types of assets, as well as on the overall utility of entrepreneurship, and therefore has a direct impact on entrepreneurial choices and the future entrepreneurial intentions of non-entrepreneurial households. Second, financial literacy releases household demand for credit and alleviates credit constraints. Financial constraints on entrepreneurial activity are the primary constraint on residents' entrepreneurial activity (Karaivanov, 2012; Weng et al., 2022). Improving financial literacy can help households understand various sources of borrowing information, credit market lending policies, and loan processes, reducing their cognitive biases and increasing their chances of successful borrowing (Akudugu et al., 2009; Cude et al., 2020), thus increasing their willingness to start a business. Third, it is beneficial for households to have the basic skills and qualities needed to carry out entrepreneurial activities (Oggero et al., 2020). Higher financial literacy enables better use of financial instruments and improves the current lack of investment opportunities, thus promoting households' participation in market investments (Van Rooij et al., 2011; Yang et al., 2022) and willingness to start a business (Rugimbana and Oseifuah, 2010; Bilal et al., 2021). Thus, by improving financial literacy, households build up long-term human capital, reduce “Poverty dependency” and engage in entrepreneurship to generate sustainable income to alleviate relative poverty. Based on this, this paper proposes the following hypothesis:

**Hypothesis 2:** Financial literacy alleviates relative poverty by reducing “Poverty dependency” and promoting household participation in entrepreneurial activities.

Financial literacy includes the ability to use financial information and then use financial literacy to plan financially, arrange for retirement and save and accumulate wealth, and is an important piece of human capital that allows individuals to manage their financial resources effectively. Individuals' financial literacy includes irrational financial behavior, such as “Risk appetite”, depending on their cultural background and work experience. In the processes of social finance, the socio-economic environment in which individuals live is changing, financial information channels are diversifying (Gudmunson and Danes, 2011; Liu et al., 2022), and risk attitudes are changing, and rational financial decisions and behaviors are being made accordingly (Jappelli and Padula, 2013; Yin and Yang, 2022). Therefore, financial literacy can improve households' subjective attitudes toward financial products that are “Risk appetite”, improve risk management, and protect against the risks of relative poverty.

Mitigating the occurrence of relative household poverty is not only about enhancing the household's ability to sustainably increase income, but also about defending against the risk of the household falling into poverty in the future (Koomson et al., 2020). In general, the larger the risk shock, the greater the likelihood that a household will fall into poverty, i.e., its vulnerability, and the more likely it is to fall into poverty when faced with a risk event. Risk attitude is seen to be an individual attribute that changes over time (Roszkowski and Davey, 2010; Baláz, 2021). On the one hand, financial literacy can change households' risk attitudes and prevent them from falling into poverty by choosing financial instruments such as insurance and credit for risk protection when facing external risks (Urrea and Maldonado, 2011; Kwon and Ban, 2021). On the other hand, through information analysis and screening of financial products, increasing social trust (Hansen, 2017) and risk-taking capacity (Hong et al., 2020), for example, by increasing households' willingness to purchase financial insurance, the insurance mechanism will work to help households diversify their risks when they are covered by insurance and other protection, thus reducing the probability of falling into poverty in the future. Based on this, this paper proposes the following hypothesis:

**Hypothesis 3:** Financial literacy alleviates relative poverty by improving “Risk appetite” and promoting household commercial insurance participation.

If households lack understanding of the loan products and lending policies, it will lead to a misunderstanding that they cannot access credit and generate demand for credit (Petrick, 2004; Howard, 2015), and there is “Information barrier”. If household members are aware of credit policies, it will facilitate their formal credit demand and access to formal credit (Akudugu et al., 2009; Pak, 2018). With the development of financial markets, households can improve their financial literacy in the process of participating in socio-economic markets, breaking down “Information barrier” and optimizing financial decision-making. Therefore, the importance of financial literacy to household financial behavior is increasingly evident, and the lack of financial literacy can be an important factor in the lack of demand for credit and demand-based credit constraints among households (Sol Murta and Miguel Gama, 2022).

This paper argues that improved financial literacy can break down “Information barrier” and facilitate households' access to credit, alleviating the incidence of relative poverty. First, improved financial literacy can help households to increase their understanding of credit market policies and procedures and reduce their cognitive biases, thereby increasing their willingness to borrow from formal financial institutions and their demand for formal credit. Families' understanding of the loan information from various channels will improve their probability of loan success (Sol Murta and Miguel Gama, 2022). It allows households to have some funds to avoid falling into poverty in case of external risk shocks or when they undertake their own financial activities. Second, increased levels of financial literacy help households to make better use of financial instruments to improve the current lack of innovation and investment opportunities, for example, households become more active in financial market investments (Van Rooij et al., 2011; Yang et al., 2022) and households gain a share of income. Thirdly, financial literacy drives household financial accumulation (Lusardi et al., 2017; Sekita et al., 2022), maintains a good credit history, and thus the availability of formal credit is likely to be better, at the same time, it promotes households' “loan application efforts”, i.e., the more financially literate they are, the more likely they are to apply to formal financial institutions. The higher the financial literacy, the more likely the household is to apply for loans from formal financial institutions, which in turn can protect the household from the risk of poverty arising from investment failure and indebtedness (Jitsuchon, 2001; Gathergood, 2012), and alleviate relative poverty. Based on this, the following hypothesis is proposed:

**Hypothesis 4:** Financial literacy alleviates relative poverty by breaking down “Information barrier” and promoting the availability of formal household credit.

## Methodology

### Sample and data collection

This paper uses data from China Household Finance Survey (CHFS) in 2017 and 2019. This survey was developed by Southwestern University of Finance and Economics to create a database to investigate the financial behavior of Chinese households. The data were collected from 29 provinces, 345 cities/counties in 2019. The head of the household, as the respondent, answer the questionnaires including items related to demographic characteristics, assets and liabilities, insurance and social security, household expenditures and income, and views on family, marriage, and community governance. The head of the household is the owner of the property of the house and is the family member who knows the most about the household's financial situation. The database can provide panel data analysis for this article.

The original data was pre-processed as follows. As CHFS survey on “financial literacy” did not cover all households, the sample of households with missing indicators was excluded, deleted the family whose head of household is under the age of 16 and over the age of 80, and the outliers of the sample were subjected to an upper and lower 5% tailing process, resulting in a sample size of 8,735 households after pre-processing.

### Variables and measures

#### Dependent variable: Relative poverty

Absolute poverty and relative poverty are the two most common types of poverty. Absolute poverty is defined as falling into absolute material deprivation because a poor household's total income is insufficient to cover basic survival expenses. It is primarily identified by establishing a minimum income or nutrition standard. However, absolute poverty theory cannot explain the persistence of poverty in developed countries (or regions), resulting in a change in the focus and difficulty of poverty governance from addressing absolute poverty to alleviating relative poverty. Relative poverty is a long-term poverty phenomenon that manifests itself primarily in a state of relative material and living conditions relative to others, and a society with abundant material resources does not eliminate the problem of relative poverty (Decerf, 2017).

The successful identification of relatively poor households in academics is still in its early stages and follows two basic paradigms, one from a welfare viewpoint, establishing a percentage of median or average income as the relative poverty level, and the other from a socioeconomic perspective (Ravallion and Chen, 2011; Chakravarty et al., 2016). The viability perspective of poverty is another relative poverty identification paradigm, which contends that relative poverty criteria should detect whether persons lack the potential to survive and socially integrate (Bourguignon and Atkinson, 2000). In Latin American nations such as Mexico and Brazil, relative poverty criteria combine income and multidimensional poverty, taking into consideration the level values of various variables such as income, education, and health. This form of identification is practical, but unlike absolute poverty eradication initiatives that focus on fundamental living stability, relative poverty governance focuses on household upward mobility and the opportunity to develop themselves so that they do not fall into poverty in the future. Based on theory of vulnerability as expected poverty (VEP), some scholars have formulated relative poverty standards from the perspective of poverty risk, which has attracted more and more attention in the field of poverty (Dang et al., 2014; Hohberg et al., 2018).

Therefore, this article measures relative poverty from two viewpoints. The first measure is to define the relative poverty level at US$3.2 per person per day consumption. In 2018, the World Bank established poverty line criteria for developing countries of $1.9 and 3.2 per person per day consumption, existing studies typically use $1.9 as the absolute poverty line, and this paper uses $3.2 as the relative poverty line for households, and after adjusting for purchasing power parity and CPI, which is RMB 4,260 per capita per year as the relative poverty line. If the surveyed household's per person per day consumption is < $3.2, the variable is set to 1, otherwise, it is set to 0. The second measure is to use the vulnerability to poverty to define the relative poverty. One popular approach considers vulnerability as expected poverty (VEP) proposed by Chaudhuri et al. (2002), i.e., the probability of a household falling into poverty in a future period. Vulnerability to poverty assesses the possibility that a household's income or level of wellbeing will fall below the poverty line if it experiences a risk shock. This indicator indicates changes in the dynamics of poverty and has significant policy consequences (Azeem et al., 2017).

It is worth noting that this paper sets a poverty line for household consumption when calculating poverty vulnerability using VEP^2^ and vulnerability cutoff based on the incidence of poverty in the current year. Table 1 reports the statistical results of household poverty and the incidence of vulnerability^3^. As can be seen from the table, although absolute poverty has improved considerably in China, the relative poverty and vulnerability rates are still high.

**Table 1:** Descriptive statistics of incidence of poverty and vulnerability to poverty.

**Areas**	**Incidence of absolute poverty**	**Incidence of relative poverty**	**Incidence of vulnerability to absolute poverty**	**Incidence of vulnerability to relative poverty**
	**$1.9**	**$3.2**	**$1.9**	**$3.2**
All areas	0.0968	0.2146	0.2437	0.4103
Rural areas	0.1349	0.3285	0.2608	0.4918
Urban areas	0.0637	0.1329	0.1942	0.3845

#### Explanatory variable: Financial literacy

Financial literacy is the ability of people to acquire basic economic information and financial ideas, manage and allocate resources in order to attain household income through financial services (Atkinson and Messy, 2011). The variable “financial literacy” has primarily been quantified in the literature by using dummy variables for household replies to financial questions in questionnaires, i.e., risk diversification, inflation, interest rate and interest compounding (Klapper et al., 2015; Grohmann et al., 2018).

This paper uses four questions involving financial knowledge and financial behavior from the Chinese Household Finance Survey (CHFS) to examine respondents' financial literacy. Two dummy variables are constructed for each question. The dummy variable corresponding to the first question is 1 if the respondent is “Extremely concerned”, “Very concerned” or “Generally concerned”, otherwise it is 0. The second and third dummy variables indicate whether the question is answered correctly (if the question is answered correctly, set the variable to 1, otherwise it is 0). The dummy variable corresponding to the fourth question is 1 if the respondent is “Project with high-risk and high-return” or “Project with slightly high-risk and slightly high-return”, otherwise it is 0. We used the iterative principal factor method to conduct factor analysis on the four questions and eight variables to produce the financial literacy indicators used in this paper (Van Rooij et al., 2011). Table 2 shows the questions designed in the questionnaire among them.

**Table 2:** The questions about financial knowledge and financial behavior to examine the financial literacy of the respondents.

**Dimensions**	**Questions**
Financial knowledge	What is your degree of concern for economic and financial information? 1. Extremely concerned 2. Very concerned 3. Generally concerned 4. Seldomly concerned 5. Not at all
	Given a 4% interest rate, how much would you have in total after 1 year if you have 100 yuan deposited? 1. Under 104 2. 104 3. Over 104 4. Cannot figure out
	With an interest rate of 5% and an inflation rate of 3%, the staff you buy with the money you have saved in the bank for 1 year is? 1. More than last year 2. The same as last year 3. Less than last year 4. Cannot figure out
Financial behavior	Which of the choice below do you want to invest most if you have adequate money? 1. Project with high-risk and high-return 2. Project with slightly high-risk and slightly high-return 3. Project with average risk and return 4. Project with slight risk and return 5. Unwilling to carry any risk 6. No idea

**Figure 2:**
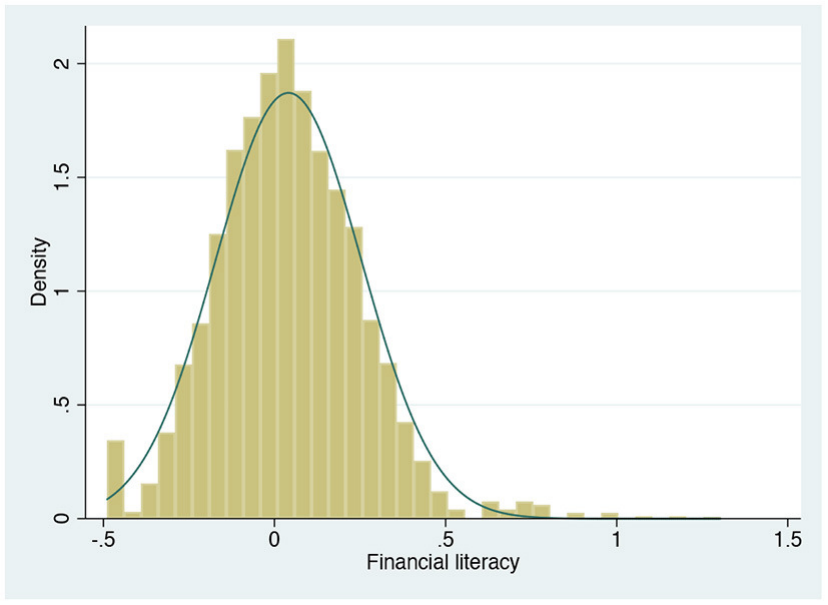
The distribution of the financial literacy index.

The distribution of the financial literacy index and the correlation between the two sub-dimensions of financial literacy are presented in Figure 2. It can be concluded that the overall financial literacy index is approximately normally distributed. Although there is some positive correlation between financial knowledge and financial behavior, the correlation coefficient between the former and the latter two is not high (correlation coefficient <0.3).

#### Control variables

Control variables are used in regression analysis to reduce the interference of other factors in estimating the primary causal effect. To analyze the influence of household financial literacy on relative poverty more effectively, the effect of other factors on relative poverty must be removed. We adjust for other factors affecting household relative poverty at three levels based on known research findings, individual characteristics, household characteristics and regional development.

Specifically, firstly, individual characteristics variables, including the gender of the household head (Gender, male = 1, female = 0), age of the household head (Age), years of education of the household head (Edu), and marital status of the household head (Marriage, married = 1, unmarried = 0). Secondly, household characteristics variables, including family scale (Family Scale), health status of household members (Health), dependency ratio (Dependency ratio), proportion of family engaged in agricultural labor (Agricultural), proportion of family engaged in non-agricultural labor (Non-agriculture), household income (Income), household net assets (Net asset), and relationship network (Network). Finally, the paper also controls for regional development variables, regional characteristics (Regional, eastern region = 1, central region = 2, and western region = 3) and regional economic development (Economic). Logarithm the following variables, household income, household net worth, and relationship network. Table 3 reports the results of the descriptive statistics of the variables.

**Table 3:** Descriptive statistics of the data used in the estimates.

**Variable**	**Obs**	**Mean**	**Standard error**	**Min**	**Max**
Poverty	10,846	0.2146	0.3285	0	1
Vulnerability	10,846	0.4103	0.2732	0	0.7385
Financial literacy	10,846	0.0436	0.0628	−0.4892	1.4032
Gender	10,846	0.5615	0.4975	0	1
Age	10,846	51.4427	14.3947	16	80
Edu	10,846	7.5329	3.9539	0	18
Marriage	10,846	0.9125	0.3250	0	1
Family Scale	10,846	3.6319	1.7506	1	8
Health	10,846	2.1082	1.9845	1	5
Dependency ratio	10,846	0.2748	0.4018	0	1
Agricultural	10,846	0.0353	0.1427	0	1
Non-agriculture	10,846	0.0672	0.1914	0	1
LN (Income)	10,846	10.8675	1.3746	8.5448	14.8652
LN (Net asset)	10,846	11.5821	2.6452	2.0958	17.7793
LN (Network)	10,846	5.4673	4.9231	0	9.3296
Regional	10,846	1.7681	0.6372	1	3
Economic	10,846	13.8256	1.4932	10.5782	15.8675

### Estimation strategy

Panel regression models always had the following advantages, which could reduce endogeneity induced by unobservable individual heterogeneity and provide more information about individuals' dynamic behavior (Baloch et al., 2020; Gallardo, 2020). To analyze that how financial literacy affects the relative poverty of households, we primarily generate the following model in this paper.

First, the panel Probit model is established as follows:


$$\begin{array}{l} Pr(poverty_{i,t}=1|finlit_{i,t},Control_{i,t})\;=\;\alpha_{0}\;+\;\alpha_{1}finlit_{i,t}\\ \;+\alpha_{2}Control_{i,t}\;+\;\mu_{i}\;+\;{\text{λ}}_{t}\;+\;\varepsilon_{i,t} \end{array}$$

Where, *poverty*_*i, t*_ is the variable of relative poverty, if the surveyed household's per person per day consumption is < $3.2, the variable is *poverty*_*i, t*_= 1, otherwise, it is set to 0. *finlit*_*i*_ indicating the household financial literacy index, *Control*_*i, t*_ would be those who affect the relative poverty but are not related to financial literacy, μ_*i*_ is individual fixed effects in household, λ_*t*_ is a period fixed effect, $\varepsilon_{it}\sim iid\left(0,\sigma^{2}\right)$ is random perturbation term, *i* is different household (*i* = 1, 2,…., 10,846), and *t* is year (*t* = 2017, 2019) and α_0_, α_1_ and α_2_ being the regression coefficients.

Second, the panel model is established as follows:


$$\begin{array}{lll} {Vulnerability}_{i,t}\; & = & \;\beta_{0}\;+\;\beta_{1}{finlit}_{i,t}\;+\;\beta_{2}{Control}_{i,t}\;+\;\rho_{i}\;+\;\nu_{t}\\ \; & + & \;\epsilon_{i,t} \end{array}$$

Where, *vulnerability*_*i, t*_ is the variable of relative poverty, the vulnerability to poverty is measured by drawing on vulnerability as expected poverty (VEP) proposed by Chaudhuri et al. (2002). See the literature for specific calculation steps (Hohberg et al., 2018). *finlit*_*i*_ indicating the household financial literacy index, *Control*_*i*_ would be those who affect the relative poverty but are not related to financial literacy, μ_*i*_ is individual fixed effects in household, λ_*t*_ is a period fixed effect, $\epsilon_{i,t}\sim iid\left(0,\sigma^{2}\right)$ is random perturbation term, *i* is different household (*i* =1, 2,…., 10,846), and *t* is year (*t* = 2017, 2019) and β_0_, β_1_, and β_2_ being the regression coefficients.

## Empirical results and analyses

### Benchmark regression results

Table 4 shows the results of the benchmark regression of household financial literacy on relative poverty. Considering that there is a certain correlation between financial literacy and variables such as household income, household net assets, and relationship network, columns (1) and (3) in Table 4 report the significant negative marginal effect of financial literacy on household relative poverty when the above control variables are not added, indicating that financial literacy helps to reduce the probability of households experiencing relative poverty. In addition, columns (2) and (4) present the complete estimates with the addition of the above control variables, the marginal effect of the financial literacy index decreases, but remains significantly negative at the 5% level and above. A comparison of the results in Table 4 shows that the significance of the coefficients of the key explanatory variables is relatively stable, and that the effect of financial literacy on relative household poverty remains significant even after controlling for other relevant variables, suggesting that household financial literacy alleviates relative poverty and Hypothesis 1 holds.

**Table 4:** Benchmark regression: household financial literacy and relative poverty.

**Variables**	**Poverty** **(1)**	**Poverty** **(2)**	**Vulnerability** **(3)**	**Vulnerability** **(4)**
Financial literacy	−0.1537***	−0.1308**	−0.4630***	−0.2831***
	(0.0299)	(0.0179)	(0.0395)	(0.0235)
LN (Income)	–	−0.0172***	–	−0.1843***
		(0.0025)	–	(0.0095)
LN (Net asset)	–	−0.0059*	–	−0.0108***
	–	(0.0014)	–	(0.0037)
LN (Network)	–	−0.0038***	–	−0.0017**
	–	(0.0029)	–	(0.0015)
Gender	0.0073**	0.0068**	0.0164**	0.0147**
	(0.0037)	(0.0032)	(0.0240)	(0.0215)
Age	0.0001	0.0001	0.0025***	0.0016***
	(0.0002)	(0.0002)	(0.0014)	(0.0012)
Edu	−0.0019***	−0.0015***	−0.0010***	−0.0008***
	(0.0054)	(0.0048)	(0.0019)	(0.0018)
Marriage	−0.0037**	−0.0026**	−0.0040**	−0.0027**
	(0.0043)	(0.0037)	(0.0042)	(0.0033)
Scale	0.0045***	0.0037***	0.1842***	0.1761***
	(0.0017)	(0.0017)	(0.0047)	(0.0046)
Health	−0.0084***	−0.0082***	−0.0173***	−0.0172***
	(0.0016)	(0.0015)	(0.0036)	(0.0036)
Raise	0.0168**	0.0139**	0.0285**	0.0279**
	(0.0174)	(0.0162)	(0.0483)	(0.0475)
Agricultural	0.0073***	0.0072***	0.3824***	0.3819***
	(0.0017)	(0.0016)	(0.0315)	(0.0310)
Non-agriculture	−0.0138***	−0.0136***	−0.0895***	−0.0892***
	(0.0174)	(0.0174)	(0.0064)	(0.0063)
Economic	−0.1498***	−0.1475***	−0.2176***	−0.2168***
	(0.0048)	(0.0046)	(0.0102)	(0.0009)
Province fixed effect	Yes	Yes	Yes	Yes
Observations	10,846	10,846	10,846	10,846

*, **, and *** denote significant at 10, 5, and 1% levels, respectively. The standard errors are reported in parentheses.

### Poverty reduction effects of financial literacy in different dimensions

Considered that there may be differences in different dimensions of household financial literacy on relative poverty alleviation, this paper further investigates the impact of household financial literacy sub-dimension dimensions on relative poverty. Firstly, columns (1) and (4) in Table 5 show that the effect of household financial knowledge on relative poverty is significantly negative at the 1% level, in other words, increased financial knowledge helps to reduce the probability of relative poverty among households. Secondly, no significant effect of household financial behavior on alleviating relative poverty is obtained from columns (2) and (5). Finally, in columns (3) and (6) of Table 5, the effects of household financial knowledge and financial behavior on relative poverty are examined simultaneously. The regression results at this point show that financial knowledge continues to have a significant negative effect, while financial behavior no longer has a significant effect. This suggests that there is some variation in the effect of the different dimensions of the household financial literacy index on relative poverty, with the effect of improving household financial knowledge on relative poverty being more significant than that of financial behavior.

**Table 5:** Distinguishing different dimensions: the impact of household financial literacy on relative poverty.

	**Poverty**	**Poverty**	**Poverty**	**Vulnerability**	**Vulnerability**	**Vulnerability**
	**(1)**	**(2)**	**(3)**	**(4)**	**(5)**	**(6)**
Financial knowledge	−0.0937***		−0.0918**	−0.2750***		−0.2428***
	(0.0175)		(0.0153)	(0.0238)		(0.0217)
Financial behavior		0.0076	−0.0048		0.0726*	−0.0635
		(0.0185)	(0.0116)		(0.0372)	(0.0329)
Control variable	Yes	Yes	Yes	Yes	Yes	Yes
Province fixed effect	Yes	Yes	Yes	Yes	Yes	Yes
Observations	10,846	10,846	10,846	10,846	10,846	10,846

*, **, and *** denote significant at 10, 5, and 1 levels, respectively. The standard errors are reported in parentheses.

### Endogeneity issue

In the above analysis, benchmark regression may have endogeneity issue due to reverse causality and omitted variables. On the one hand, threshold effect of financial markets may constrain the participation of poor households in financial markets and affect the “Learning by doing” of financial literacy, while non-poor households have more opportunities to participate in financial services and improve their financial literacy, thus there is an inverse causal relationship. On the other hand, respondents' answers to questions related to financial literacy are subjective and may be biased when administering the questionnaire. To alleviate the endogeneity issue, based on Ellis et al. (2017) and Sol Murta and Miguel Gama (2022), this paper used the “average of other households' financial literacy indices in the same community” as an instrumental variable. This is justified because respondents can improve their financial literacy by interacting with other households in the same community, and the financial literacy index of other households in the same community does not directly affect the poverty status of the household. Thus, the instrumental variable satisfies the relevance and exogeneity condition.

Table 6 reports regression results of using the instrumental variable. Column (1) and (2) shows the results of estimation using Two-stage Probit model and instrumental variable. The financial literacy index is an endogenous variable, the one-stage regression F-value and KP rk LM-value confirm that the instrumental variables are appropriate. The marginal effect of the financial literacy index increases after accounting for endogeneity, indicating that not considering endogeneity issues would underestimate the impact of financial literacy. Further, columns (3) and (4) are estimated using 2SLS, and financial literacy significantly reduces relative household poverty, with a smaller regression coefficient than when endogeneity is not considered. To increase the exogeneity of the instrumental variables, column (5) and (6) add community variables, such as community disposable income per capita, share of business and industry households, we obtain largely consistent findings. In summary, instrumental variable estimate results show that household financial literacy still significantly reduces relative poverty, while ignoring the endogeneity issue underestimates the impact of financial literacy.

**Table 6:** Estimation results of using the instrumental variable.

	**IV-Probit**		**2SLS**		**2SLS**	
	**Poverty** **(1)**	**Vulnerability** **(2)**	**Poverty** **(3)**	**Vulnerability** **(4)**	**Poverty** **(5)**	**Vulnerability** **(6)**
Financial literacy	−0.1547***	−0.3521***	−0.1630**	−0.2782***	−0.1628***	−0.2780***
	(0.0218)	(0.0217)	(0.0232)	(0.0209)	(0.0230)	(0.0208)
Controls	Yes	Yes	Yes	Yes	Yes	Yes
Community					Yes	Yes
Province fixed effect	Yes	Yes	Yes	Yes	Yes	Yes
Observations	10,846	10,846	10,846	10,846	10,846	10,846
One stage F-value	246.356***	283.175***	258.372***	325.865***	284.528**	386.154**
Wald test	157.279**	175.289**	184.319**	154.692***	175.275**	175.289***
KP rk LM-value	498.282***	448.270***	521.581***	458.596***	573.023***	476.073***

**, and *** denote significant at 5% and 1% levels, respectively. The standard errors are reported in parentheses.

### Robustness tests

To test the robustness of the benchmark regression results, firstly, the measure of relative poverty was replaced. Robustness tests were conducted using alternative relative poverty lines and vulnerability cutoff. Based on Rippin (2016), 70% of net income per capita and net household assets are used as the new relative poverty line. At the same time, we use the 50% vulnerability line for the test, and columns (1), (2), and (3) in Table 7 show that the findings obtained remain consistent with the previous ones. Secondly, the measure of financial literacy was replaced. The regression results are estimated using equal weighting method to measure the financial literacy index and the results in columns (4) and (5) indicate that the findings are consistent with those previously obtained. The paper then replaces the sample set^4^. The results in columns (6) and (7) remain unchanged, as the four municipalities are removed from the original sample. Finally, the estimation model is replaced. Given that there is a correlation between poverty and vulnerability, and that vulnerability is usually higher for households in deep poverty, this paper used Bivariate Probit model to test the poverty-reducing effects of financial literacy, and the results in columns (8) and (9) show that the basic findings remain unchanged.

**Table 7:** Robustness tests: substitution variables, sample size, and estimation model.

	**Replace dependent variable**	**Replace dependent variable**	**Replace dependent variable**	**Replace independent variable**	**Replace independent variable**	**Change sample size**	**Change sample size**	**Change estimation model**	**Change estimation model**
	**Income poverty** **(1)**	**Asset poverty** **(2)**	**Replace the line of poverty to vulnerability** **(3)**	**Poverty** **(4)**	**Vulnerability** **(5)**	**Poverty** **(6)**	**Vulnerability** **(7)**	**Poverty** **(8)**	**Vulnerability** **(9)**
Financial literacy	−0.2472***	−0.3296***	−0.1748***	−0.0956**	−0.1753***	−0.1595***	−0.3047***	−0.0364**	−0.2753***
	(0.0256)	(0.0297)	(0.0086)	(0.0065)	(0.0164)	(0.0225)	(0.0276)	(0.0162)	(0.0265)
Controls	Yes	Yes	Yes	Yes	Yes	Yes	Yes	Yes	Yes
Province fixed effect	Yes	Yes	Yes	Yes	Yes	Yes	Yes	Yes	Yes
Observations	10,846	10,846	10,846	10,846	10,846	8,146	8,146	10,846	10,846

**, and *** denote significant at 5% and 1% levels, respectively.

## Analyses of impact mechanisms

### Promoting household participation in entrepreneurial activities

According to Hypothesis 2, financial literacy alleviates relative poverty by eliminating the “Poverty dependency” effect and by promoting household participation in entrepreneurial activities. The mechanism of household participation in entrepreneurship involves two main issues: entrepreneurial activity reduces relative household poverty and financial literacy increases household willingness to start a business. This paper follows this line of thought and extends the analysis in two ways. Firstly, the sample is divided into two groups of poor and non-poor households to discuss the differential impact of financial literacy on entrepreneurship among different types of poor households. Second, dummy variables are set based on household entrepreneurship status to investigate the impact of entrepreneurial persistence on relative poverty and how financial literacy affects household entrepreneurial persistence^5^.

Using the instrumental variable from the previous section and measuring household participation in entrepreneurship based on the CHFS questionnaire “whether the household is involved in business or industry”, the variable is equal to 1 if the respondent answered “Yes” and 0 otherwise if the respondent answered “No”. In this paper, we focus on non-farm entrepreneurship. Table 8 reports the results of the estimation, where column (1) shows that household involvement in entrepreneurship helps to reduce the likelihood of poverty in the household. Columns (2), (3), and (4) show that financial literacy significantly contributes to household participation in entrepreneurship and has a greater positive effect on non-poor households. Further, columns (5) and (6) also yield that household continuation in business can significantly reduce relative poverty, with insignificant effects for initial business and exit from business, while financial literacy can significantly reduce the likelihood of household exit from business. The reason for this may be that the poverty alleviation effect of new business start-up households is insignificant compared to that of continuing households due to the short duration of the business. Exiting households also fail to improve household poverty when they exit the business due to capital, tax burden or poor business performance. In summary, financial literacy can play a positive role in alleviating relative household poverty by promoting household participation in entrepreneurship. On the one hand, financial literacy has a greater impact on promoting entrepreneurship among non-poor households, and on the other hand, financial literacy can significantly reduce the likelihood of households withdrawing from business. Therefore, Hypothesis 2 holds.

**Table 8:** Test on the impact mechanism of financial literacy on relative poverty (2SLS estimation).

**Variable**	**Poverty**	**Participation in entrepreneurship**			**Poverty**	**Exit operation**
		**Full sample**	**Poor households**	**Non-poor households**		
	**(1)**	**(2)**	**(3)**	**(4)**	**(5)**	**(6)**
Participation in Entrepreneurship	−0.1764***					
	(0.0328)					
Financial literacy		0.0634***	−0.2536	0.0816**		−0.0629**
		(0.0045)	(0.3621)	(0.0216)		(0.0362)
Continuous operation					−0.0317**	
					(0.0194)	
New operation					−0.0228	
					(0.0135)	
Exit operation					0.0164	
					(0.0144)	
Controls	Yes	Yes	Yes	Yes	Yes	Yes
Province fixed effect	Yes	Yes	Yes	Yes	Yes	Yes
Observations	9,748	9,748	1,753	9,123	9,748	9,748

**, and *** denote significant at 5% and 1% levels, respectively. The standard error of clustering at the provincial level is in parentheses.

### Commercial insurance participation

Financial literacy promotes participation in commercial insurance by improving the “Risk appetite”, which protects households from the risk of falling into poverty due to negative shocks. Financial literacy reduces the conservative and risk-averse psychological characteristics of the poor and leads to rational financial decisions, which in turn leads to increased risk tolerance through participation in commercial insurance. According to the CHFS questionnaire “whether the household buys commercial insurance”, the variable is equal to 1 if the respondent answered “Yes” and 0 otherwise if the respondent answered “No”. Using the instrumental variable from the previous section, the results are reported in Table 9, where columns (1) and (2) indicate that financial literacy significantly contributes to households' willingness to participate in commercial insurance, and that participation in commercial insurance significantly reduces household vulnerability to poverty. Hypothesis 3 holds.

**Table 9:** Test on the impact mechanism of financial literacy on relative poverty (2SLS estimation).

**Variable**	**Commercial insurance participation**	**Vulnerability**	**Formal loan channels**	**Vulnerability**
	**(1)**	**(2)**	**(3)**	**(4)**
Financial literacy	0.2467***		0.1437***	
	(0.0421)		(0.0246)	
Commercial insurance participation		−0.3146***		
		(0.0148)		
Formal loan channels				−0.0328***
				(0.0273)
Controls	Yes	Yes	Yes	Yes
Province fixed effect	Yes	Yes	Yes	Yes
Observations	9,736	9,736	9,736	9,736

*** denote significant at 1% levels. The standard error of clustering at the provincial level is in parentheses.

### The choice of lending channels

The choice of loan channel is also an important factor affecting household poverty. Poor households suffer from cognitive biases due to information barriers. Financial literacy promotes household financial accumulation, maintains good credit, promotes “Loan-seeking efforts”, thus enhances formal credit channel choice. According to the CHFS questionnaire “whether the household choose formal loan channels”, the variable is equal to 1 if the respondent answered “Yes” and 0 otherwise if the respondent answered “No”. Table 9 reports the results of regressions using instrumental variables. Column (3) and (4) shows that financial literacy has a significant positive effect on households' preference for formal loan channels, which in turn reduces household poverty vulnerability. In summary, financial literacy can mitigate household poverty vulnerability by increasing residents' risk resilience, mainly by influencing their choice to participate in formal loan channels. Hypothesis 4 holds.

### Further analysis

Robustness tests have been used above to show that financial literacy is indeed an important factor influencing households' relative poverty, and that financial literacy affects relative poverty through the institutional pathways of household participation in entrepreneurship activities, commercial insurance participation and formal loan channels. However, the following questions need further analysis. Firstly, is there a difference in the effect of financial literacy on household poverty reduction by level of financial literacy? Secondly, what are the micro effects of financial literacy on poverty alleviation by factors such as household structure, debt level and regional location? These are all questions that require further analysis.

#### Grouping of different levels of financial literacy

In this paper, households are divided into low level financial literacy group, medium level financial literacy group, medium high level financial literacy group and high-level financial literacy group according to the financial literacy index to study the impact of different levels of financial literacy of households on vulnerability to poverty. Columns (1)–(4) of Table 10 present the regression results. It suggested that financial literacy in different household groups can significantly reduce household poverty vulnerability at levels above 5%, and that the poverty reduction effect is more pronounced for households with high levels of financial literacy.

**Table 10:** Further analysis: different financial literacy groups.

	**Dependent variable: vulnerability**			
**Financial literacy**	**(1)**	**(2)**	**(3)**	**(4)**
Low level	−0.1524***			
	(0.0217)			
Medium level financial literacy		−0.2138***		
		(0.0279)		
Medium and high level			−0.2753**	
			(0.0291)	
High level				−0.3362**
				(0.0315)
Controls	Yes	Yes	Yes	Yes
Province fixed effect	Yes	Yes	Yes	Yes
Observations	2,533	4,601	2,509	1,233

**, and *** denote significant at 5% and 1% levels, respectively. The standard error of clustering at the provincial level is in parentheses.

#### Heterogeneity analysis: Different household characteristics and regional locations

This section presents a heterogeneous analysis of the microeconomic effects of financial literacy in alleviating relative household poverty in terms of household characteristics, debt level and regional location. The results of the heterogeneity analysis regressions are reported in Table 11.

**Table 11:** Heterogeneity analysis: different households characteristics and regional location.

**(A) dependent variable: poverty**					
	**Under the age of sixty**	**Age 60 and over**	**The low of debt level**	**The high of debt level**	**Eastern region**	**Central and western region**
	**(1)**	**(2)**	**(3)**	**(4)**	**(5)**	**(6)**
Financial literacy	−0.1726**	−0.0825	−0.1478***	−0.0937	−0.1513***	−0.1128***
	(0.0214)	(0.0225)	(0.0187)	(0.0329)	(0.0164)	(0.0095)
Controls	Yes	Yes	Yes	Yes	Yes	Yes
Province fixed effect	Yes	Yes	Yes	Yes	Yes	Yes
Observations	8,835	2,041	5,614	5,262	2,847	8,029
**(B) dependent variable: vulnerability**					
	**Under the age of sixty**	**Age 60 and over**	**The low debt level**	**The high** **debt level**	**Eastern region**	**Central and western region**
	**(7)**	**(8)**	**(9)**	**(10)**	**(11)**	**(12)**
Financial literacy	−0.3426***	−0.1822***	−0.3601***	−0.2124	−0.3248***	−0.2315***
	(0.0295)	(0.0133)	(0.0282)	(0.1226)	(0.0328)	(0.0213)
Controls	Yes	Yes	Yes	Yes	Yes	Yes
Province fixed effect	Yes	Yes	Yes	Yes	Yes	Yes
Observations	8,835	2,041	5,614	5,262	2,847	8,029

**, and *** denote significant at 5% and 1% levels, respectively. The standard error of clustering at the provincial level is in parentheses.

Firstly, based on the grouping of household characteristics, the full sample was divided into two groups according to the criterion of “presence of persons aged 60 years old and over in the household”. The results of the sample regressions are presented in columns (1), (2), (7), and (8) of Table 11. Except for the insignificant coefficient on financial literacy in column (2), the coefficient on financial literacy in all cases is significant at the 5% level or higher, with a negative sign, suggesting that increased financial literacy is more likely to alleviate the relative poverty of the “under the age of sixty” sample. The possible explanation for this is that the development of digital finance based on the Internet and smartphones has continued to raise the threshold of access to financial services, while older people, who mostly lack the ability to use computers and mobile phones, are more receptive to financial services than younger and middle-aged groups and are better able to enjoy the benefits of financial development.

Secondly, based on the grouping of household debt level, the entire sample was divided into two groups: including households of the low debt level and households of the high debt level, using “whether household debt level is higher than the average level of the community in which the sample is located” as the grouping criterion for measuring the level of household debt. Columns (3), (4), (9), and (10) of Table 11 show the results of the sub-sample regressions based on household indebtedness. The coefficients on financial literacy in columns (4) and (10) are not significant, while the coefficients on financial literacy in columns (3) and (9) are significant at the 1% level with a negative sign, indicating that financial literacy is more likely to reduce poverty among households with low levels of debt. Possible explanations are that groups with higher levels of indebtedness tend to have greater financing constraints, lower household income and a higher risk of households falling into poverty. At the same time, households with high levels of debt are likely to engage in more irrational economic behavior, making it difficult for them to move out of poverty quickly.

Finally, the paper uses “whether the sample household belongs to the eastern province” as the grouping criterion for regional locations, divides the full sample into “eastern region” and “central and western region”. Columns (5), (6), (11), and (12) of Table 11 show the results of the sub-samples regressions based on regional location, where the coefficients of financial literacy are all significantly negative at the 1% level, and the coefficients of the samples of “eastern region” are larger than those of the samples of “central and western region”. The regression analysis suggests that increased financial literacy is more likely to alleviate the relative poverty of households in the eastern region of China.

## Discussion

Financial literacy, an important human capital characteristic for households, is significant for alleviating relative poverty. Based on theories of poverty psychology, behavioral finance and vulnerability as expected poverty (VEP), we used panel model, an instrumental variables model, and the Probit model to investigate the impact of financial literacy on relative poverty. The empirical findings suggest that household financial literacy has the effect of alleviating poverty, which is consistent with previous findings (Xu et al., 2021), and the mechanism analysis further shows that financial literacy reduces relative poverty through promoting household participation in entrepreneurial activities, commercial insurance participation, and the choice of lending channels. The reduction poverty effect of financial literacy is more significant for “high levels of financial literacy”, “under the age of sixty”, “low levels of indebtedness”, and “households in the eastern”. Implementing multi-channel financial literacy enhancement programs to effectively improve the scope of household financial literacy, continuously improving the efficiency and quality of the household entrepreneurial environment, and actively promoting the diversification of financial products and innovation in service delivery are policy implications of our findings. The results of this paper have implications for other countries. Detailed analysis are as follows.

First, the results of benchmark regression indicate that household financial literacy can alleviate relative poverty. Unlike earlier studies, this paper seeks to explain the empirical findings using theories of poverty psychology and behavioral finance. Chronic poverty can lead to irrational social cognitive features such as negative emotions, stress, and cognitive biases, as well as the construction of self-reinforcing mechanisms of poverty, it can lead households into the poverty trap (Haushofer and Fehr, 2014). Financial literacy might have a positive impact on the subjective initiative of family members. On the one hand, it can encourage households to improve their understanding of basic financial services and work toward achieving benefits for themselves (Grohmann et al., 2018; Hasan et al., 2021). On the other hand, it can have a positive impact on family members' financial behaviors such as asset allocation decisions, financial decisions, and debt reduction (Lusardi and Peter, 2009; Lusardi and Tufano, 2015), breaking the psychological trap of poverty and increasing income and self-development capacity to alleviate relative poverty.

Second, focus on the control variables, as for household income, significant strong negative correlations appeared. The growth of the income can decrease the relative poverty, which are identical to the previous conclusions (Luo, 2022). Household income can meet the needs of family members for daily life goods, it effectively promotes the accumulation of human capital such as education and labor skills of family members, increases their own development ability, and reduces the incidence of poverty by obtaining a continuous income after participating in social labor (Shan, 2019). Household net worth and relationship networks can also alleviate relative poverty, and the building of physical and social capital in the household has a limited effect on preventing poverty but can help to reduce the probability of future poverty (Liu et al., 2019). The years of education of the household head, marital status of the household head, health status of household members, proportion of family engaged in non-agricultural labor, and regional economic development show the positive effect on household relative poverty with significance level (Zon and Muysken, 2001; Li et al., 2016; Azeem et al., 2017; Decerf, 2017). In compared to earlier research, the relative poverty rate of households is higher when the gender of the household head is male, possibly because men have a higher appetite for risk, increasing the likelihood of future household poverty, and the male do not have better saving habits than women and are weaker in resisting the risk of poverty (Almenberg and Dreber, 2015; Bannier and Neubert, 2016). Families with larger family size and higher dependency ratio can raise the economic burden on the household (Bellemare and Bloem, 2018). Households with a high proportion of agricultural labor may get less income and are more likely to fall into poverty.

Finally, the empirical findings showed that financial literacy can alleviate relative poverty through promoting household participation in entrepreneurial activities, commercial insurance participation, and the choice of lending channels. There are possible reasons for the above results. (1) According to the theory of planned behavior, households with higher financial literacy can actively participate in financial markets and various types of asset allocation and encourage household participation in entrepreneurial activities (Yang et al., 2020). Concurrently, improved financial literacy enables households to acquire the fundamental skills and literacy required to engage in entrepreneurial activities, resulting in long-term human capital accumulation (Liu M. et al., 2021), and household participation in entrepreneurship generates sustainable income to alleviate relative poverty. (2) According to theory of vulnerability as expected poverty (VEP), alleviating relative household poverty necessitates not just improving the households' ability to stabilize income, but also having the ability to resist future poverty risks. Families with higher financial literacy can change their risk attitudes and choose financial tools like insurance and loans to insulate themselves from external threats and keep their families out of poverty (Koomson et al., 2020). Simultaneously, households with higher financial literacy analyze information and evaluate financial products in boosting social trust and risk-taking abilities, as well as household willingness to purchase financial insurance (Kwon and Ban, 2021), and when these safeguards are obtained, they assist households in diversify risks and decrease the probability of future poverty. (3) According to Behrman's theoretical analysis of educational returns, financial literacy can influence the “Learning by doing” process of household participation in financial markets (Behrman et al., 2012; Lusardi et al., 2017), assisting households in understanding credit policies and lending processes in attempt to decrease cognitive biases. This may increase households' propensity to lend from formal financial institutions as well as their demand for formal credit (Rugimbana and Oseifuah, 2010; Bilal et al., 2021). Meanwhile, financial literacy drives household financial accumulation, maintains a good credit history, increases loan success chances, and with access to certain funds, households are strong enough to withstand the risk of poverty due to shocks from unknown risk factors such as investment failure and debt, alleviating relative poverty.

## Conclusion

Financial literacy significantly reduced the relative poverty of households, while financial knowledge had a more significant effect on poverty reduction. Using 2017 and 2019 China Household Finance Survey (CHFS), we analyze some mechanism effects, including that financial literacy alleviates relative poverty.

In this study, we measure the relative poverty of household from both static and dynamic perspectives. Based on The World Bank in 2018 set the poverty line criteria for developing countries, we select $3.2 per person per day of consumption for the relative poverty line. In addition, the vulnerability to poverty is measured by drawing on based on vulnerability as expected poverty (VEP) proposed by Chaudhuri et al. (2002), which reflected the dynamics of relative poverty. We then used factor analysis method to construct a household financial literacy index (including financial knowledge and financial behavior) based on the CHFS questionnaire. The empirical results show that financial literacy alleviates relative poverty through promoting household participation in entrepreneurial activities, commercial insurance participation and the choice of lending channels.

Further analysis shows that the poverty reduction effect is more pronounced for households with high levels of financial literacy. Financial literacy promotes household participation in business and industry, and continuous operation significantly reduces household relative poverty, while the effects of new operation and exit operation are not significant. The effect of financial literacy on poverty reduction is more pronounced for the households of under the age of sixty, low levels of indebtedness and in the eastern region.

The conclusions of this paper have important policy implications: first, implement a multi-channel financial literacy enhancement program to effectively increase the scope of household financial literacy. Targeting financial education at the elderly and groups with lower educational knowledge, financial literacy can be given full play to poverty reduction by increasing financial literacy education in communities and villages and building comprehensive information service platforms on the internet; enhancing their ability to allocate funds, financial planning, etc. Second, the environment for household entrepreneurship should be continuously improved to enhance the efficiency and quality of entrepreneurship. Financial support should be provided to households with a certain level of financial literacy, combined with tax and fee reductions and other means to increase households' willingness to sustain their businesses and ensure that entrepreneurial activities have a poverty-reducing effect in the long term. Third, focus on the livelihoods of vulnerable households and strengthen their risk management capacity. Improve community and village infrastructure to prevent shocks that lead to widespread exposure of households to vulnerability risks, in addition, actively promote the diversification of financial products and innovation in service delivery methods to provide vulnerable households with basic protection that can withstand certain negative risk shocks.

There are some limitations in this study. First, this study does not use a cross-country sample for empirical analysis. Financial literacy is influenced by tradition culture and educational level, and the level of developing financial literacy differs among countries. Therefore, whether the financial literacy index in this paper is applicable to other countries remains to be studied. Further research using cross-country data for the analysis modifies the bias caused by culture and tradition. Second, we should further select more appropriate instrumental variable. The average financial literacy level and welfare indicators of families will be affected by the level of regional economic development. Especially in urban families, families with similar conditions will choose to live in a community, and the exogenous nature of instrumental variables need further discussion.

## Data availability statement

The original contributions presented in the study are included in the article/supplementary material, further inquiries can be directed to the corresponding author.

## Ethics statement

Ethical review and approval were not required for the study on human participants in accordance with the local legislation and institutional requirements. Written informed consent for participation was not required for this study in accordance with the national legislation and the institutional requirements.

## Author contributions

SW: introduction, discussion, implications, and conclusion. PC: the concept, design, methods, result analysis, and paper writing. SH: editing, revising, proofreading, and language editing. All authors contributed to the article and approved the submitted version.

## Funding

This research was funded by the National Social Science Fund of China, Grant Numbers: 20STA058 and 18AJY003.

## Conflict of interest

The authors declare that the research was conducted in the absence of any commercial or financial relationships that could be construed as a potential conflict of interest.

## Publisher's note

All claims expressed in this article are solely those of the authors and do not necessarily represent those of their affiliated organizations, or those of the publisher, the editors and the reviewers. Any product that may be evaluated in this article, or claim that may be made by its manufacturer, is not guaranteed or endorsed by the publisher.

## Footnotes

^1^www.gov.cn/xinwen/2021-09/23/content_5638786.htm

^2^In this paper, we used household consumption level to measure vulnerability to poverty. On the one hand, there are often large measurement errors in income data in micro data survey, and consumption data can more accurately reflect Family Welfare (Deaton, 1989. On the other hand, using income level to calculate poverty vulnerability is difficult to find control variables, and not controlling income variables will lead to serious endogenous problems.

^3^In this paper, we used the three-step feasible generalized least squares (FGLS) to estimate the vulnerability of family poverty, see the literature for specific steps (Chaudhuri et al., 2002; Hohberg et al., 2018).

^4^Municipalities directly under the central government have strong particularity in many aspects, such as strategic positioning, spatial status and economic development. In terms of the theme of this study, previous studies have found that the economy of the four municipalities directly under the central government is relatively developed, employment opportunities are relatively sufficient, and residents' entrepreneurial motivation is inhibited (Huang and Qian, 2008). To eliminate the possible impact of the sample of municipalities directly under the central government, this study excludes the sample data of Beijing, Shanghai, Tianjin and Chongqing.

^5^According to the contents of the questionnaire CHFS 2017 and 2019, families are divided into: continuous operation (the families surveyed twice in a row participated in industrial and commercial operation), new operation (only participated in operation in 2019) and exit operation (only participated in operation in 2014).
